# The complete chloroplast genome of *Leibnitzia nepalensis* (Kunze) Kitamura, 1983 (Asteraceae, Mutisieae) and its phylogenetic analysis

**DOI:** 10.1080/23802359.2025.2463501

**Published:** 2025-02-11

**Authors:** Tianmeng Qu, Tingyu Li, Xinyu Chen, Xinyi Zheng, Hui Chen, Liang Pang, Zhixi Fu

**Affiliations:** ^a^ Key Laboratory of Land Resources Evaluation and Monitoring in Southwest, Sichuan Normal University, Ministry of Education, Chengdu, China; ^b^ College of Life Sciences, Sichuan Normal University, Chengdu, China; ^c^ Sichuan Tianshengyuan Environmental Services Co., Ltd, Chengdu, China; ^d^ Sichuan Tianshengyuan Information Technology Co., Ltd, Chengdu, China; ^e^ Sustainable Development Research Center of Resources and Environment of Western Sichuan, Sichuan Normal University, Chengdu, China

**Keywords:** *Leibnitzia*, *Leibnitzia nepalensis*, chloroplast genome, phylogenetic analysis

## Abstract

*Leibnitzia* Cass 1822 is a medicinally important herb in the Mutisieae tribe, Asteraceae family. This study reports the complete chloroplast (cp) genome of *Leibnitzia nepalensis* (Kunze) Kitamura, 1983. The quadripartite cp genome is 151,518 bp in length with 37.8% GC content, consisting of a large single copy region (83,071 bp), a small single copy region (18,365 bp), and two inverted repeat regions (25,041 bp). It encoded 130 genes, including 86 protein-coding, 36 tRNA, and 8 rRNA genes. Phylogenetic analysis revealed that the *Leibnitzia* is sister to the *Gerbera*. This study will be beneficial for the future phylogenetic and taxonomic studies of the *Leibnitzia*.

## Introduction

The genus *Leibnitzia* Cass. is mainly distributed in the Himalayas, central and eastern Asia, and North America (Nesom 1983; Hansen 1988; Baird et al. 2010; Xu et al. 2018). This genus is often utilized as a traditional medicine to clear heat, detoxify, moisten the lungs, and relieve coughs (Gu et al. 1987). Modern pharmacological study has confirmed their antibacterial properties and low toxicity (Xiao and Ding 2002). The genus comprises six species, four distributed in Asia and two from the southwestern United States to Central America (Gao and Nicholas-Hind 2011; Luo et al. 2021).

The species of *Leibnitzia nepalensis* (Kunze) Kitamura is distributed in China (Sichuan, Xizang, Yunnan), Bhutan, India, Nepal and Pakistan (Gao and Nicholas-Hind 2011, Luo et al. 2021). It has a wide distribution and is the highest-altitude (3200–4600 m) species in the genus *Leibnitzia* (Gao and Nicholas-Hind 2011), providing a significant opportunity to study its evolutionary history. The cp genome is a valuable tool for phylogenetic analyses due to its similar structures, highly conserved sequences, and stable maternal heredity (Daniell et al. 2016; Wang et al. 2022). To date, the analysis of cp genome data of *Leibnitzia anandria* has been reported (Ru et al. 2024). Since the genome data of *L. nepalensis* has not been reported previously, sequencing its genome could fill an essential gap in the phylogenetic studies of this genus and provide a reference for future taxonomic and evolutionary research.

## Materials and methods

### Sample collection, DNA extraction, and genome sequencing

**Figure 1.**
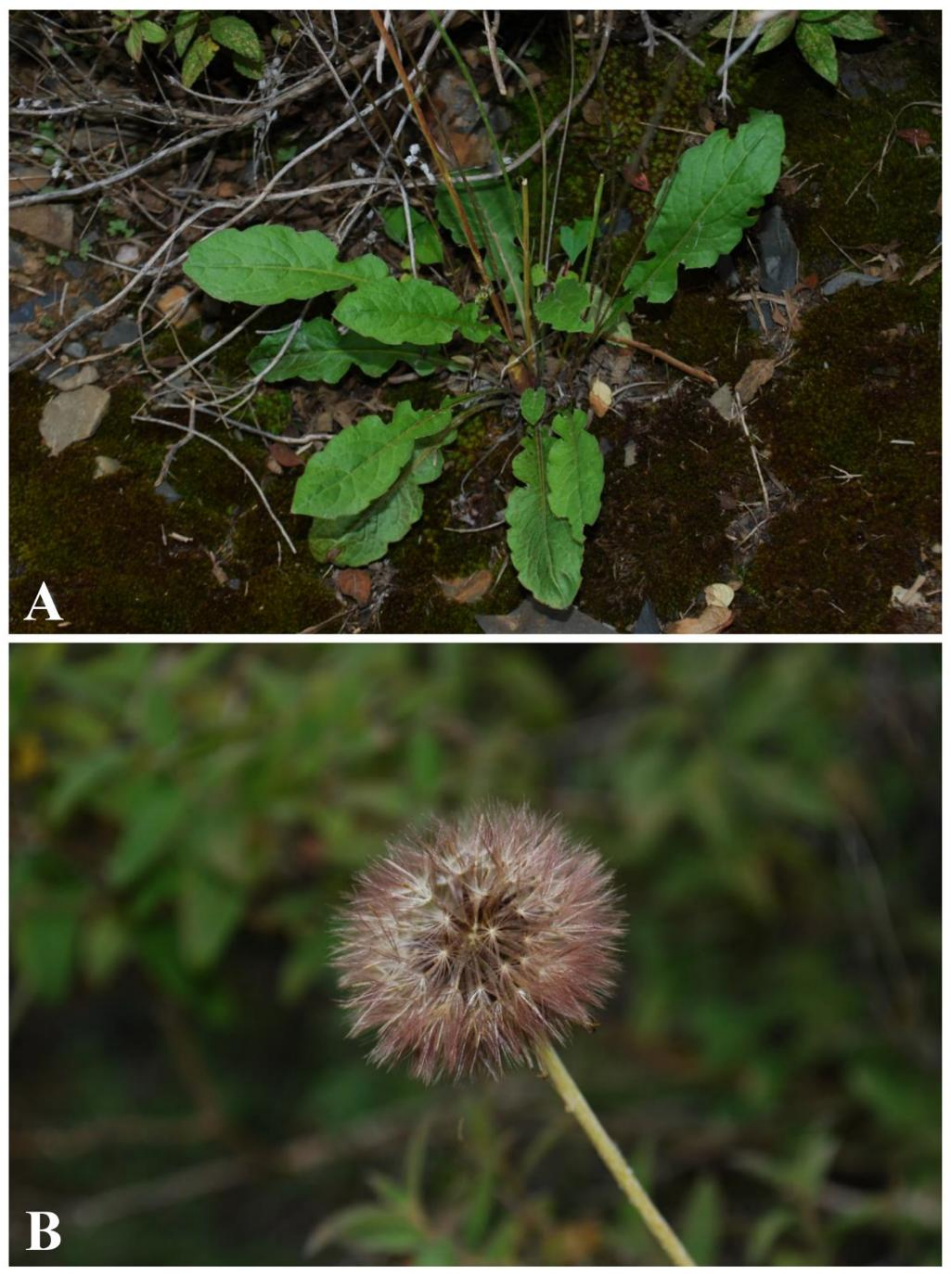
Photos of *Leibnitzia nepalensis*. (A) Habitat, (B) Inflorescence. The photo was taken by Caifei Zhang in Rumei town, Mangkang county, Changdu city, Xizang, China (98°21′3.63″E, 29°37′4.73″N). Morphological characteristics: Herbs, perennial, dimorphic; rhizome covered by marcescent leaf sheath; leaf blade ovate; capitula solitary; involucre campanulate; outer phyllaries lanceolate; florets chasmogamous; achenes (immature) terete; pappus dark purple. The photo was used with permission from Caifei Zhang and was not taken by the authors.

The species of *Leibnitzia nepalensis* was collected from Rumei town (Mangkang county, Changdu city, Xizang, China, 98°20′35.42″E, 29°36′53.36″N, alt. 3052 m) (Figure 1). The voucher specimen of TMQu no. 198 was deposited in the Herbarium of Sichuan Normal University, China (SCNU) (https://bio.sicnu.edu.cn/; contact: As. Prof. Dr. Zhixi Fu, email: fuzx2017@sicnu.edu.cn). The total genomic DNA was isolated from fresh, healthy leaves using modified CTAB methods, yielding 3808 ng (Allen et al. 2006). The quantification and evaluation of the total genomic DNA integrity were assessed using the NanoDrop 2000 Spectrophotometer and Qubit 4 Fluorometer (Thermo Fisher Scientific, Wilmington, DE, USA). After quality assessment, the DNA samples were stored at −20 °C until further processing. DNA libraries were constructed using the Illumina paired-end DNA library kit (Illumina Inc., San Diego, CA, USA), and the library preparation followed the manufacturer’s instructions. Subsequently sequenced on the Illumina HiSeq XTen platform with 150 bp paired-end reads (San Diego, CA, USA). The total read length of the raw data obtained was 3.0 Gb, and it was subjected to primary and secondary quality controls using fastp (Chen et al. 2018) to get clean data.

### Genome assembly and annotation

**Figure 2.**
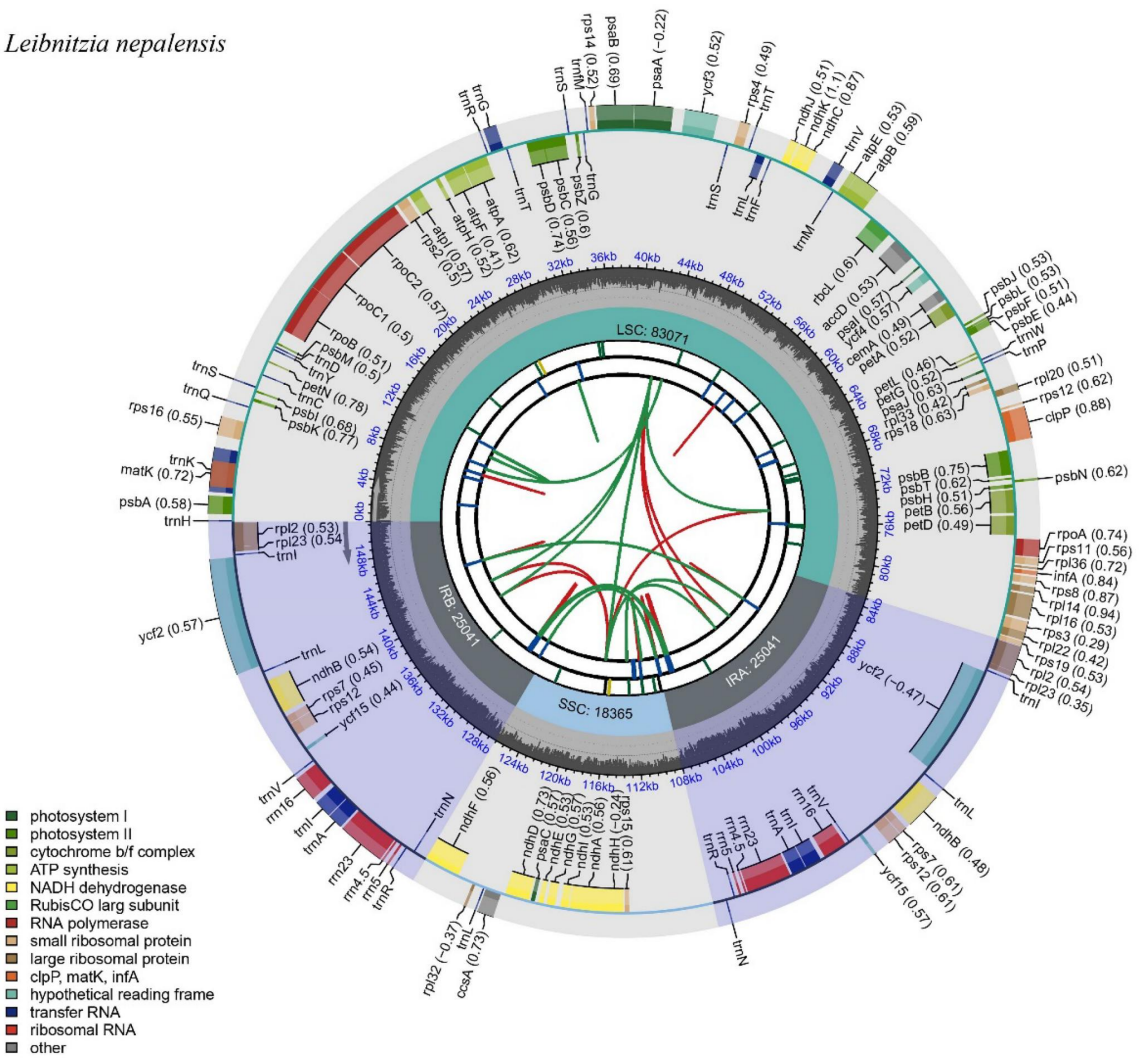
The chloroplast genome map of *Leibnitzia nepalensis*. From the center outward, the map consists of six rings. The first circle represents the forward and reverse repeats connected with red and green arcs. The second circle shows the tandem repeats marked. The third circle displays the microsatellite sequences. The fourth circle indicates the sizes of feature regions, including a large single-copy (LSC), a small single-copy (SSC), and two inverted repeats (IRa and IRb). The fifth circle exhibits the GC content. The sixth circle presents the genes with different functions.

The clean data were mapped to the reference sequences of *L. anandria* (L.) Turcz. (No. PP566209) using Bowtie2 v.2.4.5-Linux (Langmead et al. 2019). Subsequently, SAMtools v1.15-Linux (Danecek et al. 2021) was employed to retain only the reads aligned to the reference sequence for further assembly. The cp genome was assembled using default parameters with SPAdes v3.10.1 (Bankevich et al. 2012). To ensure the accuracy of the assembly, the depth of coverage was calculated by re-mapping the reads onto the assembled genome using Bowtie2 v.2.4.5-Linux (Langmead et al. 2019) and SAMtools v.1.15-Linux (Danecek et al. 2021) (Figure S1). The assembled genome was annotated with PGA software (Qu et al. 2019), followed by validation and manual refinement using Geneious R11 (Kearse et al. 2012). For visualization, the CPGview software (Liu et al. 2023) was used to generate a circular map (Figure 2) and schematic representations of cis- and trans-splicing genes (Figures S2 and S3). The complete cp genome sequence of *L. nepalensis* was submitted to the GenBank database of the NCBI under accession number OQ534531.

**Figure 3.**
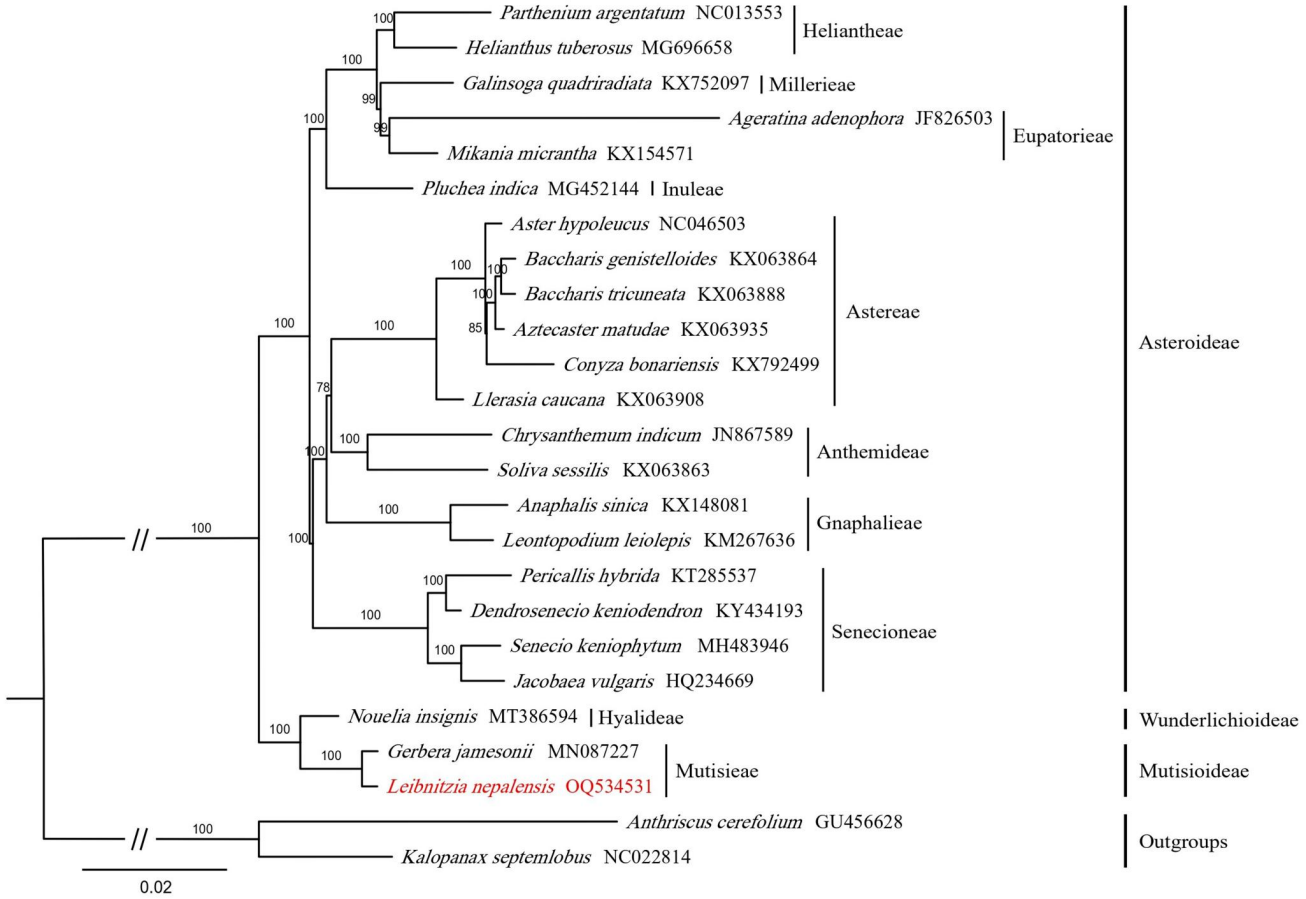
The best maximum likelihood (ML) phylogram was inferred from 25 chloroplast genomes (bootstrap values are indicated on the branches). The species marked in red is the newly sequenced species in this study. The following sequences were used: *Parthenium argentatum* NC013553 (Kumar et al. 2009), *Helianthus tuberosus* MG696658 (Xie et al. 2021), *Galinsoga quadriradiata* KX752097 (Zhang et al. 2019b), *Ageratina adenophora* JF826503 (Nie et al. 2012), *Mikania micrantha* KX154571 (Huang et al. 2016), *Pluchea indica* MG452144 (Xie et al. 2021), *Aster hypoleucus* NC046503 (Wang et al. 2019), *Baccharis genistelloides* KX063864 (Vargas et al. 2017), *Baccharis tricuneata* KX063888 (Xie et al. 2021), *Aztecaster matudae* KX063935 (Xie et al. 2021), *conyza bonariensis* KX792499 (Wang et al. 2018), *Llerasia caucana* KX063908 (Xie et al. 2021), *Chrysanthemum indicum* JN867589 (Xie et al. 2021), *Soliva sessilis* KX063863 (Xie et al. 2021), *Anaphalis sinica* KX148081 (Hoson et al. 2022), *Leontopodium leiolepis* KM267636 (Xie et al. 2021), *Pericallis hybrida* KT285537 (Siu et al. 2023), *Dendrosenecio keniodendron* KY434193 (Xie et al. 2021), *Senecio keniophytum* MH483946 (unpublished), *Jacobaea vulgaris* HQ234669 (Doorduin et al. 2011), *Nouelia insignis* MT386594 (Liu et al. 2022), *Gerbera jamesonii* MN087227 (Zhang et al. 2019a), *Leibnitzia nepalensis* OQ534531 (the newly sequenced specie), *Anthriscus cerefolium* GU456628 (Downie and Jansen 2015), *Kalopanax septemlobus* NC022814 (Li et al. 2013).

### Phylogenetic tree construction

To further explore the phylogenetic relationships between *Leibnitzia* and its related genera, RAxML version 8.2.10 (Stamatakis 2014) was utilized with 1000 bootstrap replicates based on 25 cp genomes (Table 1) to conduct the maximum-likelihood (ML) analyses. *Anthriscus cerefolium* Hoffm. (GU456628) and *Kalopanax septemlobus* (Thunb.) Koidz. (NC022814) were used as outgroups for this analysis. All sequences were aligned using MAFFT v.7.520 software with the auto pattern option (Katoh and Standley 2013), facilitating multiple sequence comparisons. A phylogenetic tree was constructed using the GTR + GAMMA model in RAxML (Stamatakis et al. 2008) with 1000 bootstrap replicates *via* the CIPRES Science Gateway portal. (https://www.phylo.org/).

**Table 1.** List of the 25 species and their GenBank accession numbers.

No.	Subfamilies	Tribes	Species	GenBank accession numbers
1	Asteroideae	Heliantheae	*Parthenium argentatum*	NC013553
2			*Helianthus tuberosus*	MG696658
3		Millerieae	*Galinsoga quadriradiata*	KX752097
4		Eupatorieae	*Ageratina adenophora*	JF826503
5			*Mikania micrantha*	KX154571
6		Inuleae	*Pluchea indica*	MG452144
7		Astereae	*Aster hypoleucus*	NC046503
8			*Baccharis genistelloides*	KX063864
9			*Baccharis tricuneata*	KX063888
10			*Aztecaster matudae*	KX063935
11			*Conyza bonariensis*	KX792499
12			*Llerasia caucana*	KX063908
13		Anthemideae	*Chrysanthemum indicum*	JN867589
14			*Soliva sessilis*	KX063863
15		Gnaphalieae	*Anaphalis sinica*	KX148081
16			*Leontopodium leiolepis*	KM267636
17		Senecioneae	*Pericallis hybrida*	KT285537
18			*Dendrosenecio keniodendron*	KY434193
19			*Senecio keniophytum*	MH483946
20			*Jacobaea vulgaris*	HQ234669
21	Wunderlichioideae	Hyalideae	*Nouelia insignis*	MT386594
22	Mutisioideae	Mutisieae	*Gerbera jamesonii*	MN087227
23			*Leibnitzia nepalensis*	OQ534531
24	Apioideae	Scandiceae	*Anthriscus cerefolium*	GU456628
25	N/A	Plerandreae	*Kalopanax septemlobus*	NC022814

## Results

The complete cp genome of *L. nepalensis* was 151,518 bp in length, featuring a typical quadripartite structure comprised of a large single-copy (LSC) region of 83,071 bp, a small single-copy (SSC) region of 18,365 bp, and a pair of inverted repeat (IR) regions, each of 25,041 bp (Figure 2). The overall GC content of the cp genome is 37.8%, with the LSC and SSC regions having GC contents of 35.87% and 31.55%, respectively. Notably, the IR regions exhibit a significantly higher GC content of 43.28%. A total of 130 unique genes were identified, including 8 rRNA, 36 tRNA, and 86 protein-coding genes. Among these, 16 genes within the IR regions are duplicated (*ndhB*, *rpl2*, *rpl23*, *rps12*, *rps7*, *rrn16*, *rrn23*, *rrn4.5*, *rrn5*, *trnA-UGC*, *trnI-CAU*, *trnI-GAU*, *trnL-CAA*, *trnN-GUU*, *trnR-ACG*, and *trnV-GAC*). Furthermore, 14 genes (*atpF*, *ndhA*, *ndhB*, *petB*, *petD*, *rpl2*, *rps16*, *rpoC1*, *trnA-UGC*, *trnG-UCC*, *trnI-GAU*, *trnK-UUU*, *trnL-UAA*, and *trnV-UAC*) are characterized by a single intron, whereas three protein-coding genes (*clpP, rps12, ycf3*) contain two introns (Figure S2). The *rps12* gene is trans-spliced, with its 5′ end situated in the LSC region and 3′ ends duplicated in each IR region (Figure S3).

The complete cp genome sequence of 23 species and the outgroup taxon (*A. cerefolium* and *K. septemlobus*) were included in the phylogenetic tree. The analyses revealed a close relationship between *Leibnitzia* and *Gerbera* L., with a high bootstrap value of 100 (Figure 3).

## Discussion and conclusion

The cp genomes of *L. nepalensis* and *L. anandria* exhibit a typical quadripartite structure (Ru et al. 2024), consistent with the chloroplast genomes of other species in the Asteraceae family (Chen et al. 2024; Li et al. 2024; Luo et al. 2024), but differ in genome size, gene content, and region lengths. *L. nepalensis* has a smaller genome (151,518 bp) than *L. anandria* (154,168 bp), with a more extended LSC region but shorter IR regions. Both genomes display similar GC content (∼37.8%) and encode comparable sets of rRNA, tRNA, and protein-coding genes, though *L. nepalensis* contains slightly fewer genes and more complex intron structures, reflecting evolutionary variations between the two species.

The phylogenetic analysis from this study aligns with previous findings (Ru et al. 2024), confirming the close relationship between *Leibnitzia* and *Gerbera* and supporting their evolutionary proximity. While Ru et al. (2024) primarily focused on the relationship between these two genera, this study extends the analysis by placing *L. nepalensis* within a broader framework that includes multiple genera and subfamilies. This approach offers more profound insights into the evolutionary dynamics between these species and provides valuable references for Asteraceae’s taxonomy and phylogenetic studies.

As *L. nepalensis* was the only representative of the genus *Leibnitzia* collected during field sampling, it became the focus of this analysis. Future studies may aim to collect additional species from the genus to enhance phylogenetic resolution and deepen our understanding of the evolutionary history of both *Leibnitzia* and the broader Asteraceae family.

## Supplementary Material

Figure supplementary captions.docx

## Data Availability

After uploading the data, the NCBI database releases two accession numbers of *L. nepalensis*. These two accession numbers contain precisely the same information sequence and author. Data are available in the NCBI GenBank at https://www.ncbi.nlm.nih.gov (accession numbers: NC073547 and OQ534531). The associated BioProject, SRA, and BioSample numbers are PRJNA1095866, SRR28538990, and SAMN40737970, respectively.
