# Supplementary material for: The complete chloroplast genome of Leibnitzia nepalensis (Kunze) Kitamura, 1983 (Asteraceae, Mutisieae) and its phylogenetic analysis
Source: Mitochondrial DNA B Resour. 2025 Feb 11;10(3):212–7. doi: 10.1080/23802359.2025.2463501 (PMC11816617; doi:10.1080/23802359.2025.2463501)
Supplement: Figure supplementary captions.docx [file TMDN_A_2463501_SM9031.docx]

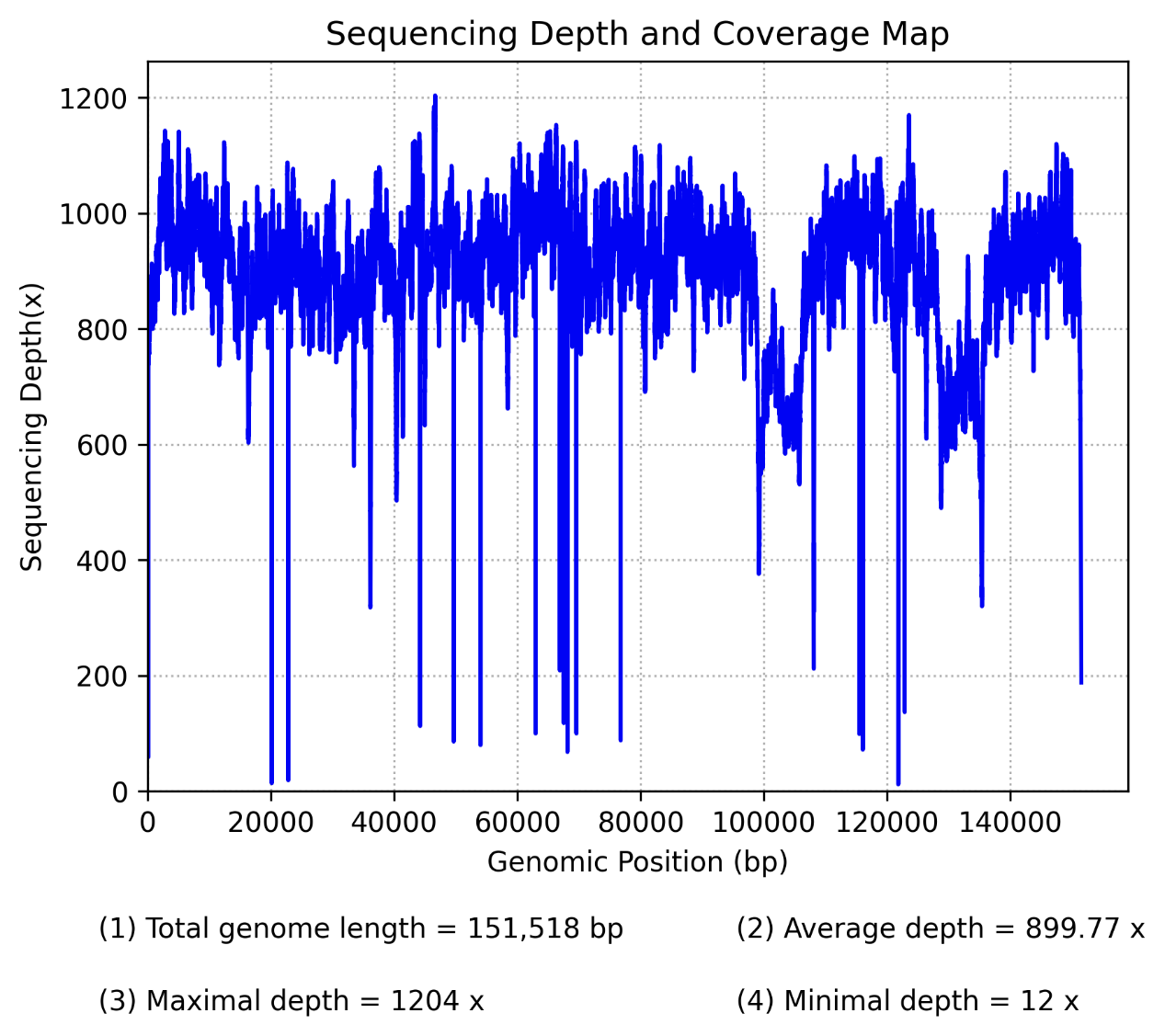


Figure S1. Overall coverage depth of the chloroplast genome assembly of *Leibnitzia nepalensis*.


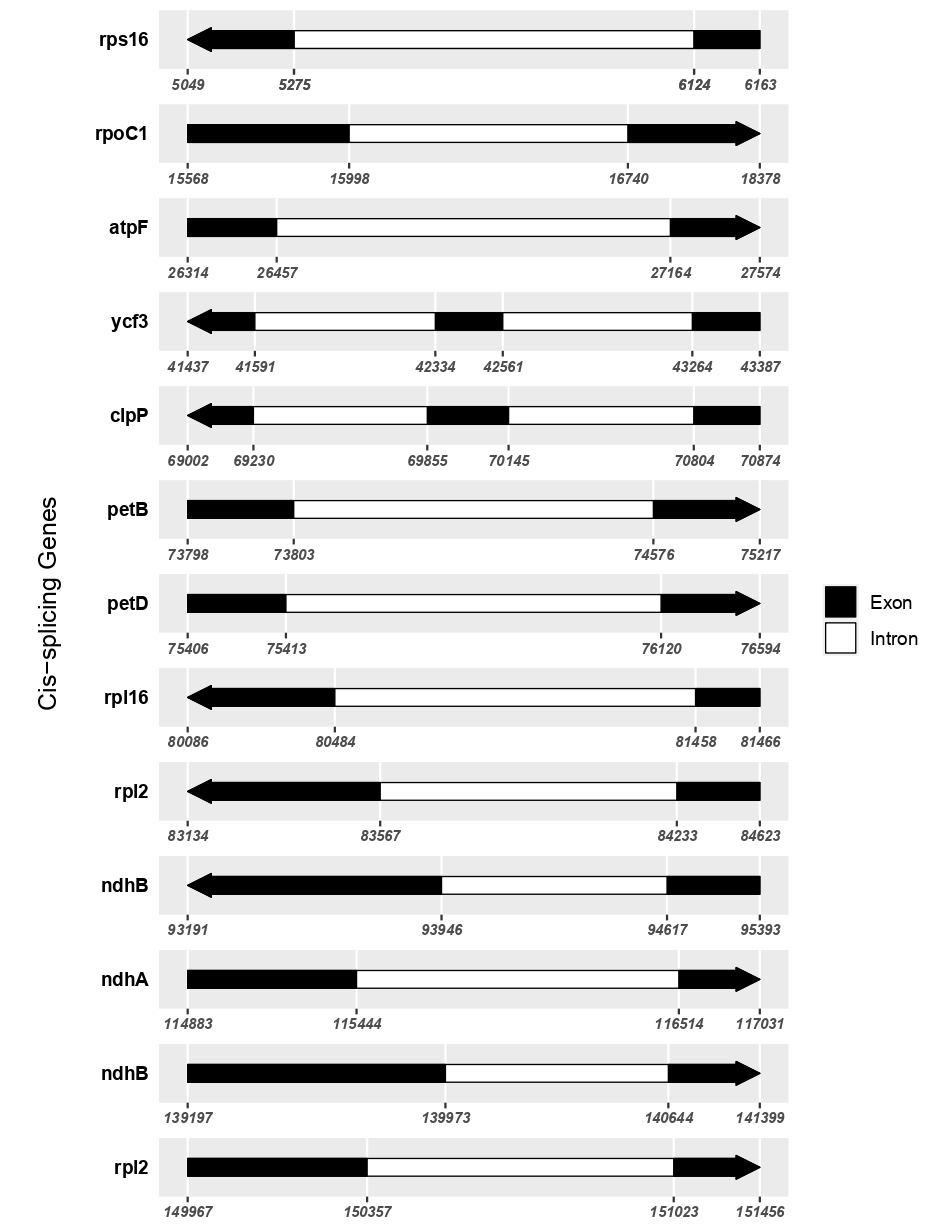


Figure S2. Schematic map of the cis-splicing genes in the *L. nepalensis* chloroplast genome.


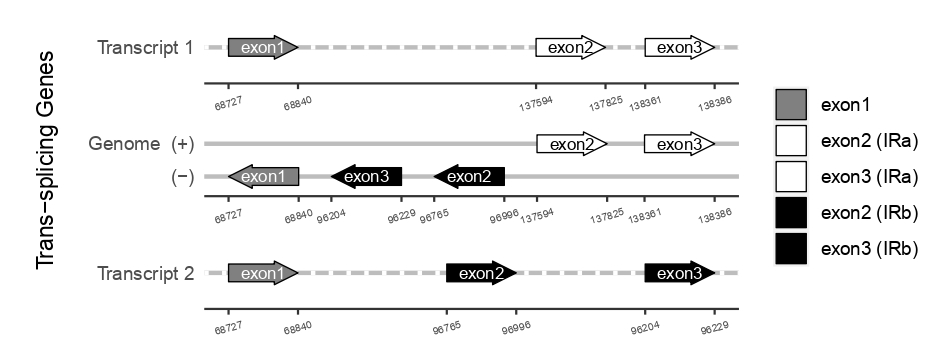


Figure S3. Schematic map of the trans-splicing gene *rps12* in the *L. nepalensis* chloroplast genome.
